# Epidemiology and Prognosis of Patients With Osteosarcoma at Different Primary Sites: A SEER Population‐Based Study

**DOI:** 10.1002/cnr2.70465

**Published:** 2026-02-10

**Authors:** Jianqun Wang, Linglong Zeng, Xiaoxia Li, Xiaozhen Fan, Zilong Yan, Hongwen Xu, Federico Canavese, Shiyu Li, Xinwang Zhi

**Affiliations:** ^1^ Department of Pediatric Orthopedics, Guangzhou Women and Children's Medical Center Guangzhou Medical University Guangzhou China; ^2^ Department of Preventive Medicine, School of Public Health Guangzhou Medical University Guangzhou China; ^3^ The Sixth Clinical Medical School Guangzhou Medical University Guangzhou China; ^4^ Department of Cardiovascular Surgery, Guangzhou Women and Children's Medical Center Guangzhou Medical University Guangzhou China; ^5^ Guangzhou Institute of Cancer Research, The Affiliated Cancer Hospital Guangzhou Medical University Guangzhou China; ^6^ Orthopedic and Traumatology Department IRCCS Istituto Giannina Gaslini Genoa Italy; ^7^ Dipartimento di Scienze Chirurgiche e Diagnostiche Integrate University of Genova Genova Italy; ^8^ Department of Microbiology and Immunology, Institute of Geriatric Immunology, School of Medicine Jinan University Guangzhou China

**Keywords:** central, osteosarcoma, peripheral, prognostic factors, survival

## Abstract

**Background:**

Osteosarcoma is a primary bone malignancy with a known bimodal age distribution. However, epidemiological patterns based on precise primary anatomical sites are not well characterized. This population‐based study analyzed the Surveillance, Epidemiology, and End Results (SEER) database to compare the incidence and clinical features of central‐site versus peripheral‐site osteosarcoma across different age groups.

**Aims:**

This study aimed to compare the incidence characteristics of peripheral and central‐site osteosarcoma (OS) and to explore the impact of different primary sites on the prognosis of patients with OS.

**Methods:**

Patients diagnosed with OS (1975–2019) were selected from the SEER databases. The different primary sites, diagnosis time, and incidence of OS were described statistically. A 1:1 propensity score matching (PSM) was used to adjust for clinical characteristics and treatment. Kaplan–Meier curves were used to compare overall survival and CSS of peripheral and central‐site OS before and after matching. Univariate and multivariate Cox models were used to investigate prognostic factors for CSS in both groups.

**Results:**

A total of 3129 patients were included (899/28.73% central‐site OS, 2166/69.22% peripheral‐site OS, 64/2.05% other‐site OS). After PSM, central‐site OS had lower overall survival and CSS than peripheral‐site OS (5‐year overall survival, 0.415 vs. 0.468; 5‐year CSS, 0.454 vs. 0.555). Multivariate analysis revealed that age (*p* = 0.010), primary site (*p* = 0.039), historical SEER stage (regional, *p* = 0.012; distant, *p* < 0.001), histologic grade (grade III, *p* = 0.014; grade IV, *p* = 0.009), surgery (*p* < 0.001), and radiotherapy (*p* = 0.005) were significant factors for CSS. Subgroup analyses adjusting for these factors showed better CSS in peripheral‐site OS patients.

**Conclusions:**

The incidence of central‐site OS is lower than that of peripheral‐site OS, while the prognosis of patients with peripheral‐site OS is more favorable than that of patients with central‐site OS. Surgical intervention is a cornerstone in the management of OS and is effective for both central‐site and peripheral‐site OS.

## 1 Introduction

Osteosarcoma (OS) is a relatively rare malignant bone tumor. However, it is the most common primary bone cancer in children and adolescents, with an incidence rate of approximately 0.3 cases per 100 000 individuals [1, 2]. The epidemiology, distribution and prognosis of OS vary depending on the primary site [1, 2, 3].

Notably, a European prospective study of 113 patients with primary malignant OS found that the five‐year survival rate was higher for OS of the limbs (66.9%) than for OS of the pelvis (44%) or other axial bones (55%) [4]. However, some studies have reported that the primary site of OS does not significantly affect survival outcomes [5, 6]. This discrepancy may be due to inadequate and inconsistent classification of the primary sites.

Considering the potential for complete resection and the tumor behavior, we categorized OS into central‐site and peripheral‐site OS based on the initial site of onset. Our study systematically compared the epidemiology, characteristics, and long‐term prognosis of OS among different sites. Furthermore, based on the Osteosarcoma Surveillance, Epidemiology, and End Results (SEER) database (https://seer.cancer.gov/), which serves as a comprehensive OS database, this study analyzed and compared the impact of the initial site of OS, including the type of tissue from which they originated, on their frequency and long‐term prognosis. Additionally, the prognostic implications of OS with different primary sites in different risk factors were also investigated.

## 2 Materials and Methods

### 2.1 Patient Selection

The data on patients with OS used in this study were obtained from the SEER database (https://seer.cancer.gov/‐ Incidence‐SEER Research Plus Data, 8 registries, Nov 2021 Sub [1975–2019]; version 8.4.0).

All patients with OS [Adolescents and Young Adults Site Recode 2020 Revision Site Group: 4.1 Osteosarcoma] were included. The exclusion criteria were defined as follows: (1) individuals diagnosed with OS without a confirmed pathological diagnosis; (2) individuals diagnosed with a primary cancer other than OS as their first malignancy; and (3) individuals for whom survival data were unavailable.

OS cases were classified by primary site into three groups: peripheral site (extremities), central site (skull, mandible, vertebral column, ribs, and pelvic bones), and other site OS, neither peripheral nor central site.

### 2.2 Statistical Analysis

Data visualization included pie charts, histograms, and trend graphs to analyze the distribution of OS cases by primary site. The annual age‐adjusted incidence of OS by year of diagnosis and cancer‐specific survival (CSS) were accessed using SEER*Stat software version 8.4.3 (National Cancer Institute (NCI), Bethesda, Maryland, USA). A log‐rank test was further performed to assess the statistical significance of the differences between the groups.

To minimize differences in covariates between central‐site and peripheral‐site OS cases, propensity score matching (PSM) was performed at a 1:1 ratio with a caliper of 0.02 based on the closest propensity score on the logit scale. Matched covariates included age, sex, race, laterality, SEER historical stage, histologic grade, surgery, radiation therapy, chemotherapy, and the total number of in situ malignancies. Kaplan–Meier curves were used to compare overall survival and CSS between central‐site and peripheral‐site OS groups, focusing on the 5‐year survival rate.

Univariate and multivariate Cox regression analyses were conducted to identify variables associated with prognosis and to determine independent prognostic factors that significantly affect prognosis. Variables with *p*‐values less than 0.05 in the univariate analyses were chosen as candidates for the multivariable Cox regression analysis. The “Forward Wald” stepwise procedure was used to construct the multivariable Cox regression model.

All statistical analyses were performed using R software version 4.3.1 (Foundation for Statistical Computing, Vienna, Austria), and SPSS 25.0 (IBM Corporation, Armonk, NY, USA). A two‐tailed *p*‐value less than 0.05 was considered statistically significant.

## 3 Results

### 3.1 Study Population

**FIGURE 1:**
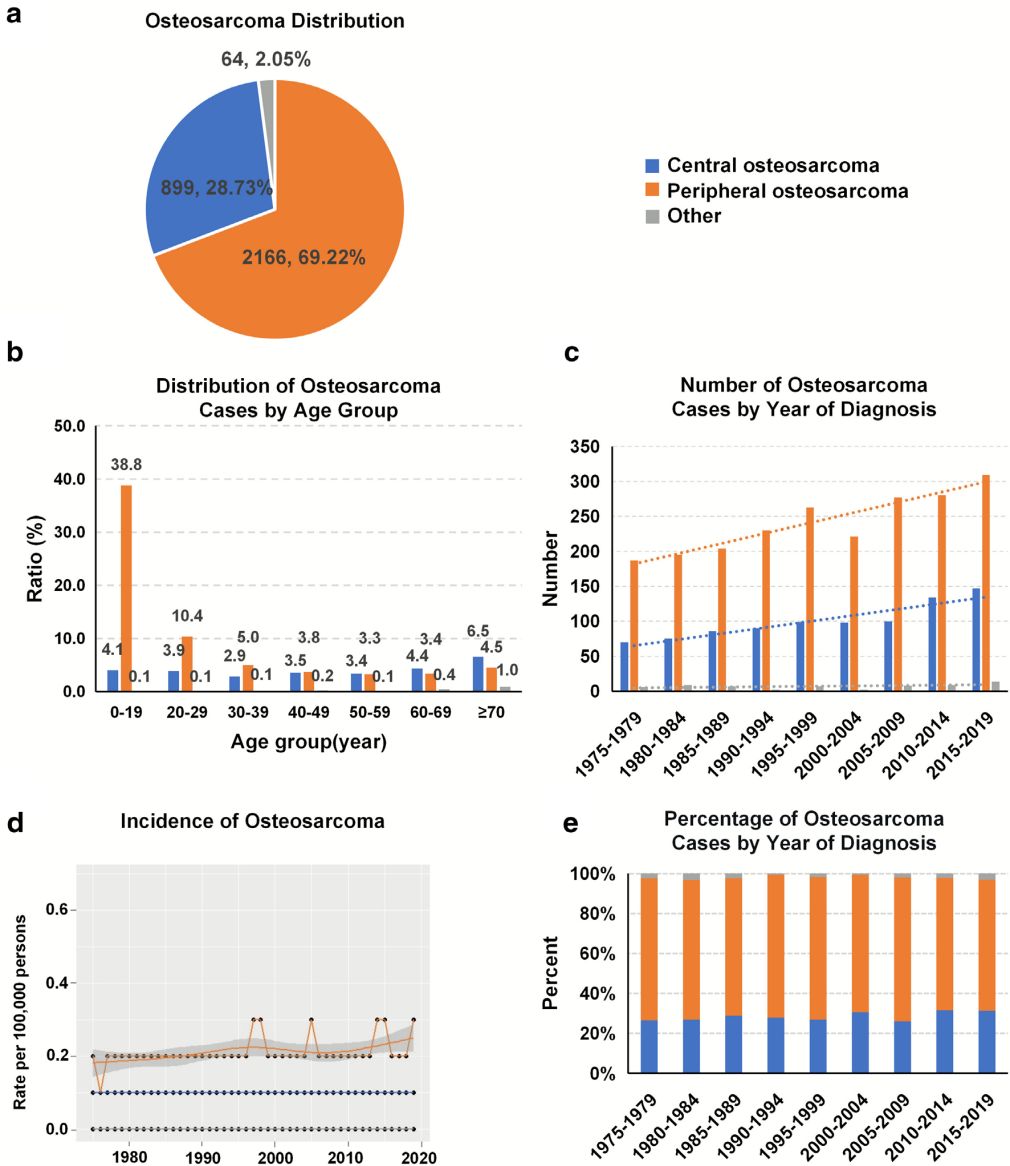
The distribution of osteosarcoma cases by site and the incidence rate of osteosarcoma in the SEER database from 1975 to 2019. (a) Distribution of osteosarcoma in different primary sites; (b) Number of osteosarcoma cases by age group; (c) Number of osteosarcoma cases in different primary sites from 1975 to 2019; (d) Incidence rate of osteosarcoma in different primary sites from 1975 to 2019; (e) Percentage of osteosarcoma in different primary sites from 1975 to 2019.

A total of 3129 patients (1694/54.14% men and 1435/45.86% women) with OS (mean age: 33.70 ± 23.76 years) were included in the SEER database from 1975 to 2019. Among these patients, peripheral‐site OS accounted for the largest proportion at 69.22% (*n* = 2166), followed by central‐site OS at 28.73% (*n* = 899), and other‐site OS at 2.05% (*n* = 64); the mean ages of patients with peripheral‐site, central‐site, and other‐site OS were 26.83 ± 20.65 years, 48.18 ± 22.83 years, and 62.80 ± 21.82 years, respectively (Figure 1a). The distribution of osteosarcoma (OS) by age and primary site is detailed in Figure 1b. The 0–19 years group represented the largest proportion (42.9%), with peripheral‐site OS (38.83%) far more common than central‐site OS (4.09%). Peripheral‐site OS prevalence declined with age, from a peak in the youngest group to 4.54% in those aged ≥ 70 years. The number of cases of central‐site OS increased from 70 (26.62%, 1975–1979) to 147 cases (31.28%, 2015–2019), and the number of cases of peripheral‐site OS increased from 187 (71.1%, 1975–1979) to 309 cases (65.74%, 2015–2019). The number of cases of other‐site OS increased from 6 (2.28%, 1975–1979) to 14 cases (2.98%, 2015–2019) (Figure 1c). However, the incidence of central‐site, peripheral‐site and other‐site OS remained stable at 1/1 000 000, 2/1 000 000 and < 0.1/1 000 000 (Figure 1d). The proportions of central‐site, peripheral‐site and other‐site OS showed stability within the ranges of 25%–32%, 65%–72%, and 0.5%–4% (Figure 1e).

After screening according to our exclusion criteria, we included 2582 eligible patients with OS in the analysis. The overall five‐year CSS for this population was 59.8% (95% CI: 57.8%–61.8%). However, there was a significant difference in the 5‐year CSS between central‐site OS and peripheral‐site OS, which were 46.7% (95% CI: 42.5%–50.8%) and 63.8% (95% CI: 61.6%–66.0%), respectively (*p* < 0.001). These results suggest a significant correlation between the primary site of OS and its incidence and prognosis (Table 1).

**TABLE 1:** Specific mortality by primary site group.

Time	Overall				Central‐site OS				Peripheral‐site OS			
	*n*	CSS	SE	95% Cl	*n*	CSS	SE	95% CI	*n*	CSS	SE	95% CI
12 months	2582	85.8%	0.7%	84.4%–87.1%	610	74.5%	1.8%	70.8%–77.9%	1972	89.3%	0.7%	87.8%–90.6%
24 months	2582	72.9%	0.9%	71.1%–74.6%	610	60.1%	2.0%	56.8%–64.0%	1972	76.8%	1.0%	74.9%–78.7%
36 months	2582	66.2%	1.0%	64.3%–68.1%	610	53.6%	2.1%	49.4%–57.6%	1972	70.1%	1.1%	67.9%–72.1%
48 months	2582	62.2%	1.0%	60.2%–64.1%	610	48.9%	2.1%	44.7%–53.0%	1972	66.2%	1.1%	64.0%–68.4%
60 months	2582	59.8%	1.0%	57.8%–61.8%	610	46.7%	2.1%	42.5%–50.8%	1972	63.8%	1.1%	61.6%–66.0%
120 months	2582	55.7%	1.0%	53.7%–57.7%	610	42.0%	2.2%	37.7%–46.2%	1972	59.9%	1.2%	57.5%–62.1%

Abbreviations: Cl, confidence interval; CSS, cancer‐specific survival; *n*, number; OS, osteosarcoma; SE, standard error.

**FIGURE 2:**
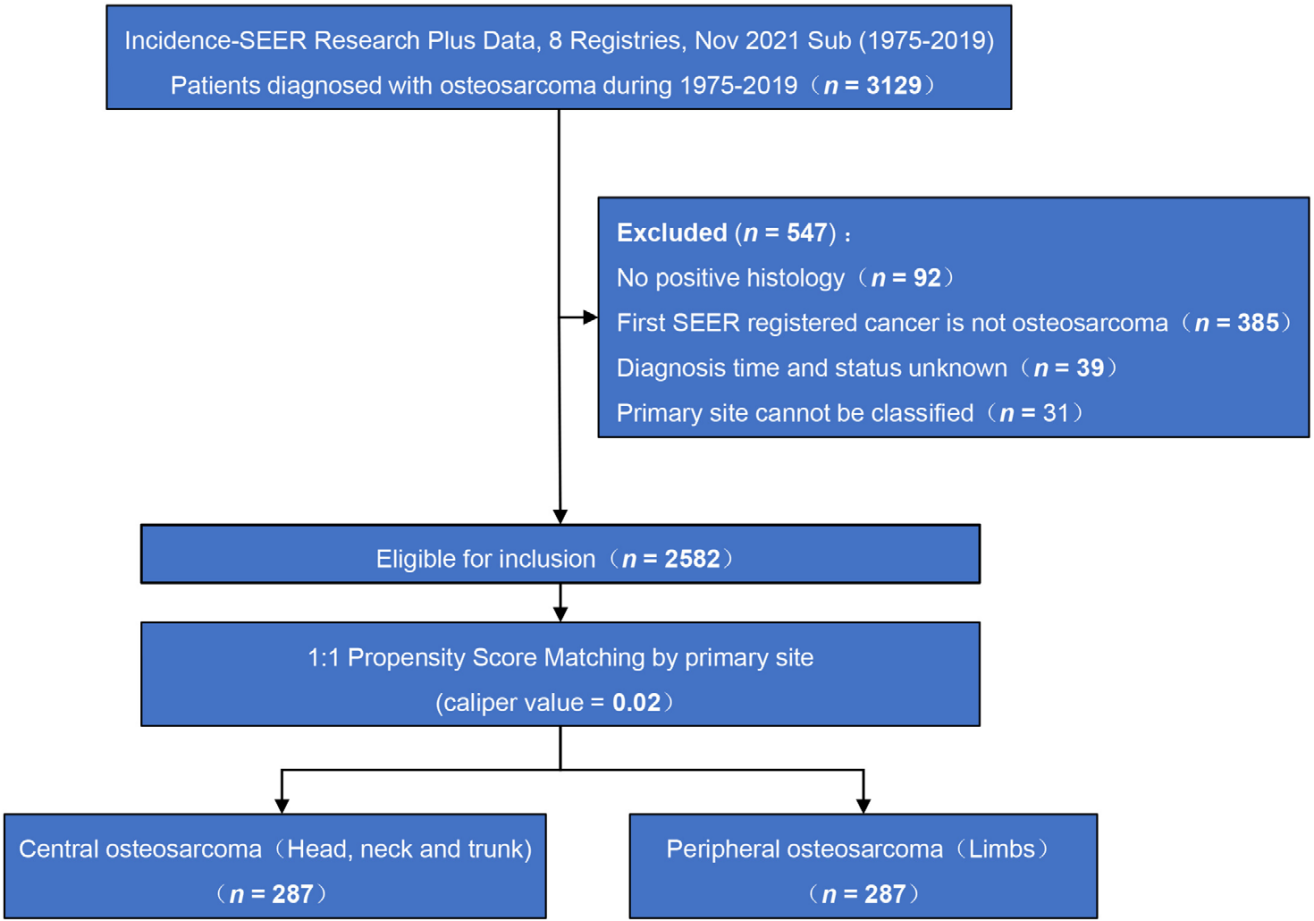
Flowchart of osteosarcoma case selection and propensity score matching.

To address baseline imbalances, we selected 287 pairs of OS cases using PSM (Figure 2). This reduced bias and improved comparability between the central and peripheral osteosarcoma groups, allowing a reliable assessment of their impact on incidence and prognosis (Table 2).

**TABLE 2:** Patients characteristics.

Characteristic	Baseline					PSM^a^			
	Overall	Central‐site OS	Peripheral‐site OS	*p*		Overall	Central‐site OS	Peripheral‐site OS	*p*
	(*n* = 2582)	(*n* = 610)	(*n* = 1972)			(*n* = 574)	(*n* = 287)	(*n* = 287)	
Age group (years)									
≤ 18	1186 (45.9%)	90 (14.8%)	1096 (55.6%)	< 0.001		132 (23.0%)	64 (22.3%)	68 (23.7%)	0.692
> 18	1396 (54.1%)	520 (85.2%)	876 (44.4%)			442 (77.0%)	223 (77.7%)	219 (76.3%)	
Sex									
Female	1149 (44.5%)	302 (49.5%)	847 (43.0%)	0.004		287 (50.0%)	142 (49.5%)	145 (50.5%)	0.802
Male	1433 (55.5%)	308 (50.5%)	1125 (57.0%)			287 (50.0%)	145 (50.5%)	142 (49.5%)	
Race									
Black	291 (11.3%)	61 (10.0%)	230 (11.7%)	0.003		64 (11.1%)	24 (8.4%)	40 (13.9%)	0.007
White	1984 (76.8%)	498 (81.6%)	1486 (75.4%)			451 (78.6%)	241 (84.0%)	210 (73.2%)	
Other	307 (11.9%)	51 (8.4%)	256 (13.0%)			59 (10.3%)	22 (7.7%)	37 (12.9%)	
Laterality									
Left	1062 (41.1%)	121 (19.8%)	941 (47.7%)	< 0.001		243 (42.3%)	111 (38.7%)	132 (46.0%)	0.199
Right	1072 (41.5%)	113 (18.5%)	959 (48.6%)			195 (34.0%)	105 (36.6%)	90 (31.4%)	
Other	448 (17.4%)	376 (61.6%)	72 (3.7%)			136 (23.7%)	71 (24.7%)	65 (22.6%)	
SEER historic stage									
Distant	402 (15.6%)	111 (18.2%)	291 (14.8%)	0.003		118 (20.6%)	57 (19.9%)	61 (21.3%)	0.751
Localized	820 (31.8%)	158 (25.9%)	662 (33.6%)			165 (28.7%)	78 (27.2%)	87 (30.3%)	
Regional	928 (35.9%)	231 (37.9%)	697 (35.3%)			184 (32.1%)	96 (33.4%)	88 (30.7%)	
Unknown	432 (16.7%)	110 (18.0%)	322 (16.3%)			107 (18.6%)	56 (19.5%)	51 (17.8%)	
Histologic stage									
Grade I	103 (4.0%)	27 (4.4%)	76 (3.9%)	< 0.001		31 (5.4%)	15 (5.2%)	16 (5.6%)	0.236
Grade II	150 (5.8%)	49 (8.0%)	101 (5.1%)			26 (4.5%)	10 (3.5%)	16 (5.6%)	
Grade III	431 (16.7%)	117 (19.2%)	314 (15.9%)			116 (20.2%)	50 (17.4%)	66 (23.0%)	
Grade IV	715 (27.7%)	131 (21.5%)	584 (29.6%)			126 (22.0%)	63 (22.0%)	63 (22.0%)	
Unknown	1183 (45.8%)	286 (46.9%)	897 (45.5%)			275 (47.9%)	149 (51.9%)	126 (43.9%)	
Surgery									
None/unknown	421 (16.3%)	154 (25.2%)	267 (13.5%)	< 0.001		154 (26.8%)	76 (26.5%)	78 (27.2%)	0.851
Yes	2161 (83.7%)	456 (74.8%)	1705 (86.5%)			420 (73.2%)	211 (73.5%)	209 (72.8%)	
Radiotherapy									
None/unknown	2298 (89.0%)	439 (72.0%)	1859 (94.3%)	< 0.001		461 (80.3%)	236 (82.2%)	225 (78.4%)	0.248
Yes	284 (11.0%)	171 (28.0%)	113 (5.7%)			113 (19.7%)	51 (17.8%)	62 (21.6%)	
Chemotherapy									
No/unknown	670 (25.9%)	265 (43.4%)	405 (20.5%)	< 0.001		232 (40.4%)	117 (40.8%)	115 (40.1%)	0.865
Yes	1912 (74.1%)	345 (56.6%)	1567 (79.5%)			342 (59.6%)	170 (59.2%)	172 (59.9%)	

Abbreviation: OS, osteosarcoma.

^a^ PSM, propensity score matching.

### 3.2 Survival Analysis

**FIGURE 3:**
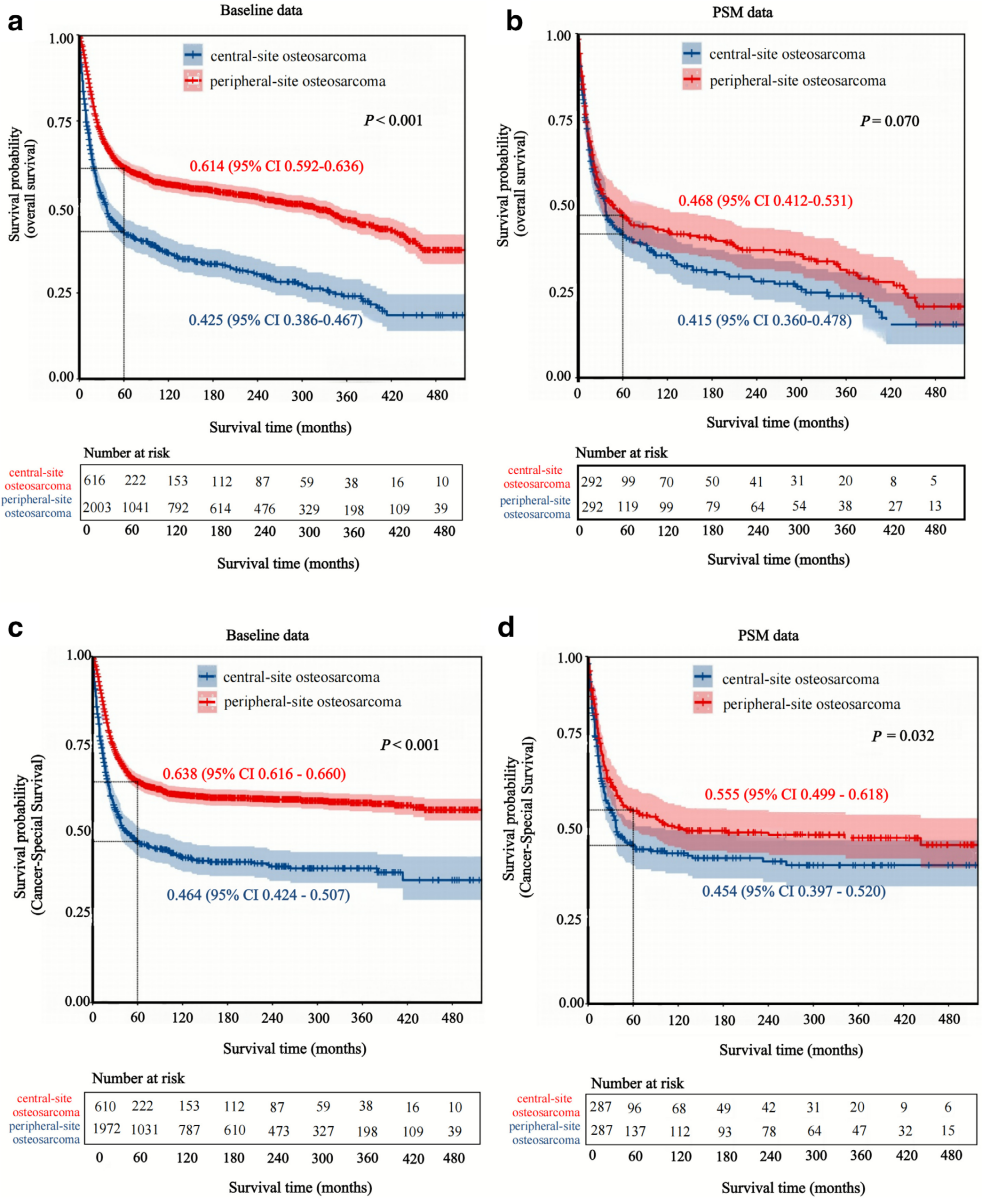
Kaplan–Meier survival curves for central‐site vs. peripheral‐site osteosarcoma before and after PSM analysis; (a) overall survival curves by primary site before PSM (*p* < 0.001); (b) overall survival curves by primary site after PSM (*p* = 0.070); (c) cancer‐specific survival curves by primary site before PSM (*p* < 0.001); (d) cancer‐specific survival curves by primary site after PSM (*p* < 0.032).

The Kaplan–Meier analysis revealed that patients with peripheral‐site OS had better 5‐year overall survival and CSS compared to those with central‐site OS before PSM (5‐year overall survival: 0.614 vs. 0.425, *p* < 0.001; 5‐year CSS: 0.638 vs. 0.464, *p* < 0.001). After PSM, the survival differences in the matched population are substantially attenuated with overlap of the 95% CI on the matched curves. In particular, no significant difference in overall mortality was observed between peripheral‐site and central‐site OS (5‐year overall survival: 0.468 vs. 0.415, *p* = 0.070). However, a significant difference in CSS remained, despite some overlap in the 95% CIs (5‐year CSS: 0.555 vs. 0.454, *p* = 0.032) (Figure 3).

### 3.3 Prognostic Factors Associated With the CSS of Osteosarcoma

**FIGURE 4:**
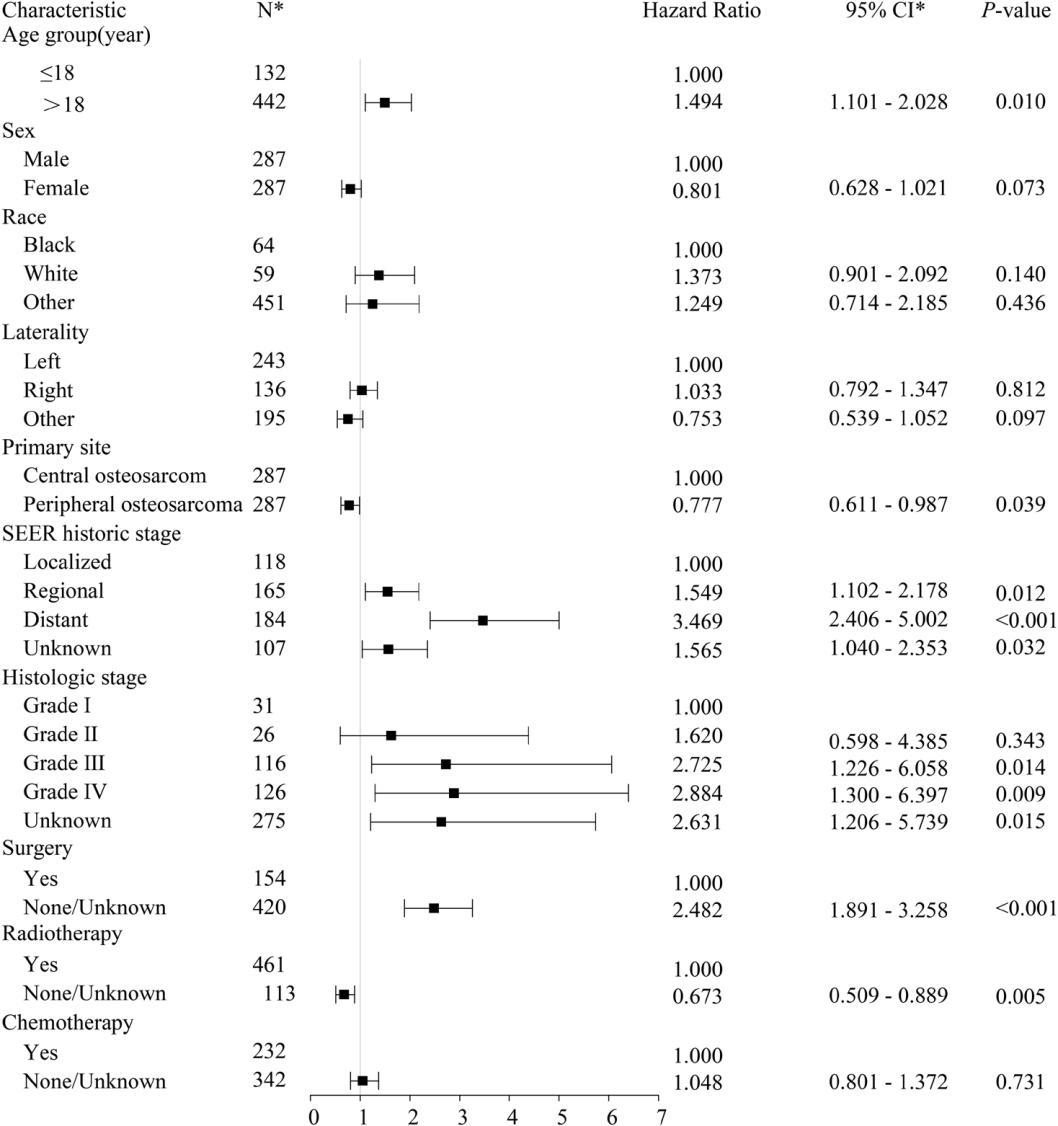
Multivariate Cox regression analyses for predicting the mortality in patients with osteosarcoma. Cl*, confidence interval; N*, number.

We examined several factors to identify prognostic indicators for CSS in OS patients in the univariate Cox regression analyses. Primary site, gender, SEER historical stage, histologic grade, and surgical treatment or radiotherapy were all found to be significantly correlated with CSS in univariate Cox regression analysis (Table 3). Further multivariable Cox analysis revealed that primary site, age, SEER historic stage, histologic grade, and surgical treatment or radiotherapy were independent prognostic indicators for CSS (Figure 4).

**TABLE 3:** Univariable regression analyses for predicting the mortality of the patients with OS.

	Hazard ratio	95% CI	*p*
Age group (years)			
≤ 18	Reference		
> 18	1.201	0.909–1.585	0.197
Sex			
Male	Reference		
Female	0.740	0.587–0.933	**0.011**
Race			
Black	Reference		
White	1.503	0.995–2.269	0.053
Other	1.112	0.639–1.936	0.708
Laterality			
Left	Reference		
Right	1.171	0.903–1.518	0.233
Other	0.738	0.541–1.007	0.055
Primary site			
Central‐site OS	Reference		
Peripheral‐site OS	0.778	0.617–0.981	**0.034**
SEER historic stage			
Localized	Reference		
Regional	1.631	1.173–2.268	**0.004**
Distant	5.010	3.591–6.969	**< 0.001**
Unknown	1.945	1.310–2.889	**0.001**
Histologic stage			
Grade I	Reference		
Grade II	1.682	0.626–4.516	0.302
Grade III	2.917	1.329–6.403	**0.008**
Grade IV	3.795	1.747–8.243	**< 0.001**
Unknown	3.425	1.603–7.318	**0.001**
Surgery			
Yes	Reference		
None/unknown	3.105	2.442–3.947	**< 0.001**
Radiotherapy			
Yes	Reference		
None/unknown	0.474	0.364–0.618	**< 0.001**
Chemotherapy			
Yes	Reference		
None/unknown	0.855	0.673–1.086	0.199

*Note:* Bold values indicate a statistically significant difference with *p* < 0.05.

Abbreviations: Cl, confidence interval; OS, osteosarcoma.

Patients with peripheral‐site OS had more favorable CSS than those with central OS (HR 0.777; 95% CI: 0.611–0.987; *p* = 0.039). On the other hand, age over 18 years (HR 1.494; 95% CI: 1.101–2.028; *p* = 0.010), regional metastasis (HR 1.549; 95% CI: 1.102–2.178; *p* = 0.012), distant metastasis (HR 3.469; 95% CI: 2.406–5.002; *p* < 0.001), higher histologic grade (Grade III: HR 2.725; 95% CI: 1.226–6.058; *p* = 0.014; Grade IV: HR 2.884; 95% CI: 1.300–6.397; *p* = 0.009), and no surgical treatment (HR 2.482; 95% CI: 1.891–3.258; *p* < 0.001) were associated with a negative impact on CSS. Interestingly, patients who receive radiotherapy had worse CSS (HR 0.673; 95% CI: 0.509–0.889; *p* = 0.005) (Figure 4).

**FIGURE 5:**
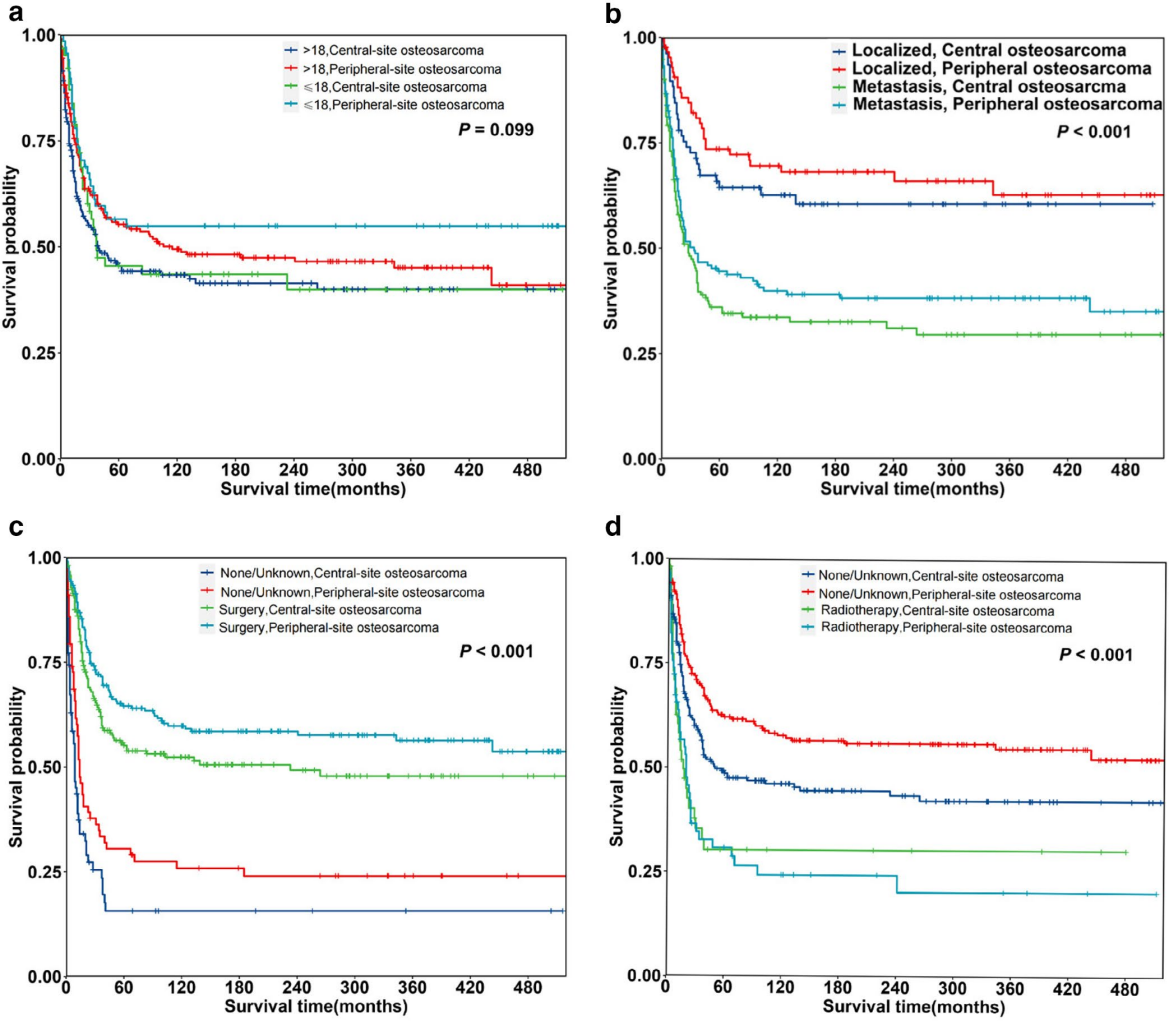
Kaplan–Meier survival curves for osteosarcoma by clinical characteristics; (a) survival by age group and primary site, showing no significant difference between age groups (*p* = 0.099); (b) survival by SEER historical stage, with significant differences between localized and metastatic osteosarcoma (*p* < 0.001); (c) survival by surgery status, showing better survival for patients who underwent surgery (*p* < 0.001); (d) survival by radiotherapy status, showing worse survival for patients who received radiotherapy (*p* < 0.001).

Additionally, we compared CSS of central‐site and peripheral‐site OS by age group. Regardless of age, the primary site of OS (central‐site vs. peripheral‐site) did not significantly affect 5‐year CSS (*p* = 0.099; Figure 5a). However, patients with peripheral‐site OS had better 5‐year CSS than those with central‐site OS for all stages including localized and metastatic malignancy (*p* < 0.001; Figure 5b). Patients with peripheral‐site OS had a higher 5‐year CSS than those with central‐site OS, regardless of whether they underwent surgery (*p* < 0.001; Figure 5c). Regardless of whether patients had central‐site or peripheral‐site OS, those who were known to have received radiotherapy had worse 5‐year CSS (*p* < 0.001; Figure 5d).

## 4 Discussion

This study provides a comprehensive comparison of the epidemiologic characteristics and long‐term prognosis of central and peripheral site OS. In contrast, other studies often focus on comparisons between specific sites, such as pelvic, spine, upper extremity, lower extremity, head and neck OS [7, 8, 9, 10]. In particular, our study found that the incidence of OS in patients from the SEER database varies with the primary site, and the prognosis for patients with peripheral‐site OS is more favorable than for those with central‐site OS.

Overall, the central‐site OS is lower than that of peripheral‐site OS, which has been supported by several other studies [11, 12]. Fernandes et al. found 1927 cases (95.9%) of primary OS in the extremities and 82 cases (4.1%) in the trunk [6]. It has been hypothesized that such a difference may be related to the development and anatomical characteristics of different bones.

The prognosis of OS varies according to the primary site, with peripheral‐site OS having a better long‐term prognosis than central‐site OS. The 5‐year survival rate of peripheral‐site OS from the SEER database was higher than that of central‐site OS (63.8% vs. 46.7%). In fact, the overall survival and CSS results of peripheral‐site OS were better than that of central‐site OS. Ferrari et al. found that the 5‐year CSS of peripheral‐site OS versus central‐site OS was 38.8% and 28.3%, respectively, which is consistent with the conclusion of the current study [12]. Ottesen et al. also found that primary OS in axial bone (skull and facial bones, mandible, vertebral column, pelvic bones, sacrum, coccyx, and associated joints) was a predictive factor for poor patient survival [13]. However, some studies have shown that the primary site of OS does not affect survival [5, 6, 14]. The following factors may have led to the controversy: (1) Previous research mainly focused on osteosarcomas of different bone types and how they affect prognosis; however, they did not consider osteosarcomas originating from other tissues [15, 16]; (2) cranial OS were not included [17, 18]; (3) the number of patients was relatively small [19, 20].

The higher risk of progression and mortality associated with central‐site OS is primarily due to the challenges of achieving complete surgical resection and the more complex histology characterized by a richer blood supply [16, 21]. Additionally, the most common clinical indicator of OS is a mass, allowing earlier detection and diagnosis of peripheral‐site OS [22].

The results of our prognostic factor analysis for OS at central and peripheral sites showed that primary site, age, historical SEER stage, histological grade, surgery, and radiotherapy were significant prognostic factors. Previous studies have reported similar results, suggesting that these factors may lead to different prognoses for primary OS at central and peripheral sites [13, 23, 24, 25]. Our findings indicate that chemotherapy has no significant impact on the prognosis of survival for OS patients; however, this result should be interpreted with caution due to notable limitations. First, only approximately 40% of the cases in the SEER database had documented receipt of standard chemotherapy. Second, chemotherapy combined with surgical resection remains the established standard of care for OS in clinical practice [1].

While our study indicates similar prognoses for peripheral and central OS in adults and children, previous studies have reported better outcomes for peripheral‐site OS compared to central‐site OS, particularly in children and adolescents. Smeland et al. found that children or adolescents with OS have a better prognosis than adults [26]. However, an epidemiological study of 3017 Korean adolescent patients with OS showed that the 5‐year overall survival rate was 61%, which was negatively correlated with age and consistent with the results of a Japanese bone and soft tissue tumor registry study [10]. This may be because younger patients have fewer malignant tumor cells, fewer invasive OS cells, or greater treatment tolerance.

Surgery is a protective factor for OS [27]. A statistically significant difference in CSS was observed between patients with peripheral‐site OS who underwent surgery and those with central‐site OS. Furthermore, our preliminary analysis indicates a higher overall survival rate in the surgical group. However, we were unable to identify the specific treatments received by patients classified as “None/Unknown” due to limitations in the data structure of the SEER database. Several studies have shown that surgery is crucial in the early management of OS, primarily through limb salvage procedures and amputation [12, 28, 29, 30]. However, because the SEER database does not collect data on the specific type of surgery performed, we were unable to analyze the prognostic impact of different surgical procedures.

Although the Cox regression analysis in this study suggests that patients who received radiotherapy had a worse prognosis, regardless of whether the primary site was central or peripheral, limitations of the SEER database prevent us from definitively ruling out the effectiveness of radiotherapy for patients with osteosarcoma. To some extent, radiotherapy may contribute to improved survival [27]. Radiotherapy is often used in cases where complete resection of the tumor has not been achieved [22, 31]. In cases where surgery alone cannot completely remove the tumor, for example, if the tumor is in bones such as the buttocks or face, radiotherapy may be a beneficial option [22, 32].

We encountered several limitations in the analysis of our results. First, there is the potential for selection bias in case selection and incomplete data collection. Second, the SEER database lacks information on medical history, specific treatment regimens (preoperative or postoperative chemo‐radiotherapy, specific chemotherapeutic agents), histopathologic response to preoperative chemotherapy, patients' underlying disease, economic status, and drug tolerance. Third, subgroup analysis of outcomes by treatment type was unavailable. Lastly, the reliability of the treatment variables in the SEER database is limited. The absence of this information hinders a comprehensive assessment of treatment effectiveness. Despite these limitations, our study has the advantages of a large dataset and a long 40‐year follow‐up, making the results more credible than those of current research on OS, which is limited in both data quantity and follow‐up time.

In future research, we suggest that further in‐depth studies should be conducted on the molecular biological characteristics and behavior of OS at the central and peripheral sites. This will help elucidate the mechanisms behind the occurrence and development of OS. It will also provide a more accurate basis for individualized treatment and prognostic evaluation.

## 5 Conclusion

The incidence of central‐site OS is lower than that of peripheral‐site OS, while the prognosis of patients with peripheral‐site OS is more favorable than that of patients with central‐site OS. Surgical intervention is a cornerstone in the management of OS and is effective for both central‐site and peripheral‐site OS.

## Author Contributions

Conceptualization: Jianqun Wang and Xinwang Zhi. Data curation: Linglong Zeng and Xiaoxia Li. Formal analysis: Linglong Zeng and Xiaoxia Li. Funding acquisition: Jianqun Wang and Xinwang Zhi. Investigation: Linglong Zeng, Xiaoxia Li, and Xiaozhen Fan. Methodology: Jianqun Wang, Linglong Zeng, Xiaoxia Li, and Xiaozhen Fan. Project administration: Shiyu Li, Federico Canavese, and Xinwang Zhi. Resources: Xinwang Zhi. Software: Linglong Zeng, Xiaoxia Li, and Xiaozhen Fan. Supervision: Jianqun Wang and Xinwang Zhi. Validation: Shiyu Li, Federico Canavese, and Xinwang Zhi. Visualization: Linglong Zeng, Xiaoxia Li, and Xinwang Zhi. Writing – original draft: Linglong Zeng and Xiaoxia Li. Writing – review and editing: Shiyu Li, Federico Canavese, and Xinwang Zhi. All authors have read and agreed to the published version of the manuscript.

## Funding

This work was supported by the Guangzhou Basic Research Plan City school (hospital) co‐funded project: 2024A0310898, Guangzhou Health Science and Technology General Guidance Project: 20241A011044, Guangzhou Basic and Applied Basic Research Project: 2024A04J3858, National Natural science Foundation of china: 82303244 and Guangzhou Municipal Health commission Youth Talent Training Program: 20261A031035.

## Ethics Statement

All primary studies in this analysis obtained ethical clearance from their institutional review boards and obtained informed consent from participants. This study used publicly accessible summary‐level data for secondary analysis. Since the study did not use individual‐level data or involve direct interaction with human subjects, no additional ethical approval was required.

## Consent

The authors have nothing to report.

## Conflicts of Interest

The authors declare no conflicts of interest.

## Data Availability

The data that support the findings of this study are available from the corresponding author upon reasonable request.
